# Supplementary material for: CT-Based Radiomics and Deep Learning for BRCA Mutation and Progression-Free Survival Prediction in Ovarian Cancer Using a Multicentric Dataset
Source: Cancers (Basel). 2022 May 31;14(11):2739. doi: 10.3390/cancers14112739 (PMC9179845; doi:10.3390/cancers14112739)
Supplement: Supplementary file 1 [file cancers-14-02739-s001.zip › cancers-1682383-supplementary.pdf]

Supplementary material

Table S1

The selected radiomic features are classified based on the feature family: intensity-based statistical features (F_stat.), textural features based on the grey level co-occurrence matrix (F_cm), textural features based on the grey level run length matrix (F_rlm), textural features based on the grey level size zone matrix (F_szm).

The full names of the selected radiomic features are available in the IBSI reference manual as follows:

- *Statistical features*

'F_stat.kurt'	Kurtosis
'F_stat.min'	Minimum intensity
'F_stat.max'	Maximum intensity
'F_stat.range'	Intensity range

- *Textural features based on the grey level co-occurrence matrix*

'F_cm_merged.joint.max'	Joint maximum, with merging by slice
'F_cm.diff.avg'	Difference average
'F_cm.diff.entr'	Difference entropy
'F_cm.inv.diff.norm'	Normalised inverse difference
'F_cm.inv.var'	Inverse variance
'F_cm.corr'	Correlation
'F_cm_merged.joint.entr'	Joint entropy, with merging by slice
'F_cm_2.5D.energy'	Energy (angular second moment), with merging by direction
'F_cm_2.5D.corr'	Correlation, with merging by direction
'F_cm.2.5Dmerged.info.corr.1'	First measure of information theoretic correlation, with full merging

- *Textural features based on the grey level run length matrix*

'F_rlm.r.perc'	Run percentage
'F_rlm.glnu.norm'	Normalised grey level non-uniformity

- *Textural features based on the grey level size zone matrix*

'F_szm.lze'	Large zone emphasis
'F_szm.z.perc'	Zone percentage
'F_szm.lzhge'	Large zone high grey level emphasis
